# Brain Gliomas and Ollier Disease: Molecular Findings as Predictive Risk Factors?

**DOI:** 10.3390/cancers14143464

**Published:** 2022-07-16

**Authors:** Sergio Corvino, Giuseppe Mariniello, Giuseppe Corazzelli, Raduan Ahmed Franca, Marialaura Del Basso De Caro, Rosa Della Monica, Lorenzo Chiariotti, Francesco Maiuri

**Affiliations:** 1 Department of Neurosciences, Reproductive and Odontostomatological Sciences, Neurosurgical Clinic, School of Medicine, University of Naples “Federico II”, 80131 Naples, Italy; giumarin@unina.it (G.M.); giucoraz@gmail.com (G.C.); frmaiuri@unina.it (F.M.); 2 Department of Advanced Biomedical Sciences, Section of Pathology, School of Medicine, University of Naples “Federico II”, 80131 Naples, Italy; raduanahmed.franca@unina.it (R.A.F.); marialaura.delbasso@unina.it (M.D.B.D.C.); 3 Department of Molecular Medicine and Medical Biotechnology, School of Medicine, University of Naples “Federico II”, 80131 Naples, Italy; dellamonica@ceinge.unina.it (R.D.M.); lorenzo.chiariotti@unina.it (L.C.)

**Keywords:** Ollier disease, anaplastic astrocytoma, enchondromatosis

## Abstract

**Simple Summary:**

As reported in the recent Classification of the Tumors of the Central Nervous System (2021), the role of molecular markers in CNS tumor diagnosis and classification, and accordingly in the decision making of the therapeutic process, has become even more decisive. In this scenario, we performed an accurate literature review of patients with Ollier disease harboring brain gliomas, disclosing only thirty cases; our own case was also included. Most of the reported cases belong to the pre-molecular era. We found a strong relationship between an IDH mutation in the enchondroma of patients with Ollier disease and the occurrence of brain gliomas; this finding may allow an early diagnosis of brain glioma in these patients, detecting the tumor when still small and silent, thus allowing the neurosurgeon, oncologist, and radiotherapist to plan the best management.

**Abstract:**

**Background:** Ollier disease (OD) is a rare nonhereditary type of dyschondroplasia characterized by multiple enchondromas, with typical onset in the first decade of life. Surgery is the only curative treatment for primary disease and its complications. Patients with OD are at risk of malignant transformation of enchondromas and of occurrence of other neoplasms. **Methods:** A wide literature review disclosed thirty cases of glioma associated with OD, most of them belonging to the pre-molecular era. Our own case was also included. Demographic, clinical, pathologic, molecular, management, and outcome data were analyzed and compared to those of sporadic gliomas. **Results:** Gliomas associated with OD more frequently occur at younger age, present higher rates of multicentric lesions (49%), brainstem localizations (29%), and significantly lower rates of glioblastomas (7%) histotype. The IDH1 R132H mutation was detected in 80% of gliomas of OD patients and simultaneously in enchondromas and gliomas in 100% of cases. **Conclusions:** The molecular data suggest a higher risk of occurrence of glioma in patients with enchondromas harboring the IDH1 R132H mutation than those with the IDH1 R132C mutation. Thus, we suggest considering the IDH1 R132H mutation in enchondromas of patients with OD as a predictive risk factor of occurrence of glioma.

## 1. Background

Ollier disease (OD) is a rare nonhereditary type of dyschondroplasia, with a prevalence of 1:100,000 per year, characterized by enchondromatosis and areas of dysplastic cartilage, caused by a developmental anomaly in enchondral ossification mainly involving long tubular bones, such as the femur, tibia, and fibula, but also flat bones such as the pelvis and small bones of the hands and feet, with asymmetric distribution [1,2]. Intracranial localizations involve the skull base, mainly the sphenoid bone, foramen lacerum, and parasellar region [1,2].

The clinical presentation is characterized by local pain, bone swelling, asymmetric shortening of a body extremity and palpable bony masses on a finger, with or without pathologic fractures and/or bone deformity.

Ollier disease can occur with other mesenchymal tumors, such as angiosarcomas, osteosarcomas, and thyroid adenomas, or with non-mesenchymal tumors, such as leukemia [3], ovarian carcinoma [4], and central nervous system gliomas [5,6,7,8,9,10,11,12,13,14,15,16,17,18,19,20,21,22,23,24,25,26,27]. The prevalence of malignancies in OD is about 53% [28], whereas that of sarcomatous degeneration of enchondromas ranges from 20 to 50% [1,10,29]. Surgery represents the only effective treatment, allowing for tumor removal and treatment of complications, such as fractures, growth defects, and neurological symptoms.

The literature review disclosed only 30 cases of gliomas associated with Ollier disease [5,6,7,9,10,11,12,13,14,15,16,17,18,19,20,21,22,23,24,25,26,27,30], most of them belonging to the pre-molecular era. 

We describe a further case of a young man with OD, since he was an infant, who developed an intracranial multifocal fibrillary diffuse astrocytoma when he was 27 years old and was treated with a biopsy followed by chemotherapy, achieving improvement of neurological symptoms and partial tumor regression, and, six years later, underwent microsurgical resection after tumor progression. Furthermore, we compare this case with the literature review on this topic and discuss the role of molecular markers. 

## 2. Methods

A Medline search up to January 2022 in PubMed online electronic database was made using the following key phrases: “Ollier disease and brain gliomas”, “Ollier disease”, “Ollier disease and brain tumors”, “Ollier disease and astrocytoma”, “Ollier disease and oligodendroglioma”, and “Ollier disease and glioblastoma”. The inclusion criteria were surgical series, reviews, and case reports in English, as well as papers written in other languages, but including an abstract in English. All reported cases of brain gliomas associated with Ollier disease meeting the inclusion criteria, including our personal case, were enrolled. 

Demographic, clinical, pathologic, molecular, management, and outcome data were analyzed and compared to those of sporadic gliomas.

The consent of our patients was not required by our institution, for this study, since all data were sufficiently anonymized.

## 3. Results

### 3.1. Case Description

**Figure 1:**
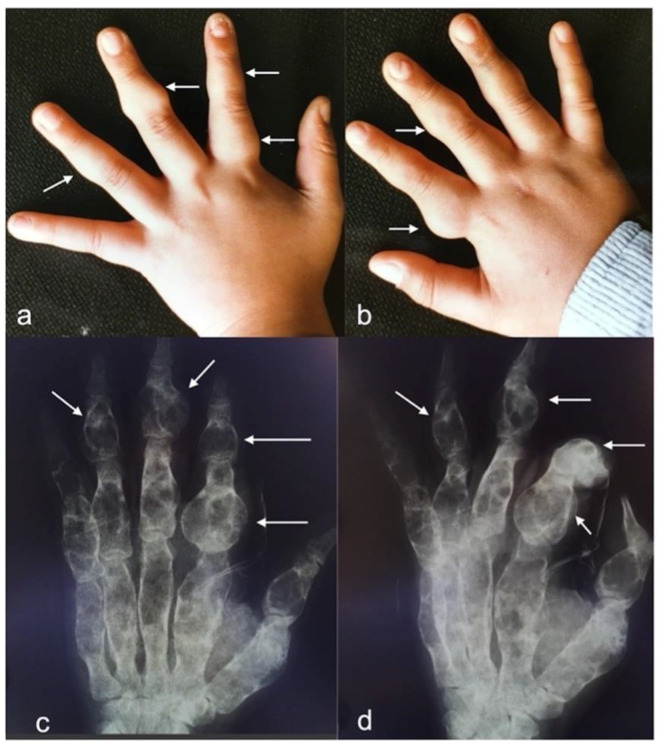
**Photograph of hands.** (**a**,**b**) Left and right hands with multiple palpable bony nodules (white arrows). (**c**,**d**) **X-rays of left hand** showing multiple enchondromas at the proximal phalanx of the second finger and intermediate phalanxes of second, third, and fourth fingers.

A 33-year-old man was observed in November 2021 in our neurosurgical unit for the management of a brain glioma. When he was two years old he was found to be affected by Ollier disease, mainly localized in both hands. Thus, at the age of ten years he underwent resection of multiple enchondromas of the fingers (Figure 1). 

In 2017, because of the occurrence of an epileptic seizure, the patient underwent a brain MRI, which disclosed small (<2 cm) intracerebral lesions in both frontal lobes. A stereotactic biopsy of the left frontal lesion, performed at another institution, was in favor of a WHO grade II IDH-1 (R132-H) mutant fibrillary diffuse astrocytoma, with proliferation index Ki67-MIB1 1%. The adjuvant treatment with temozolomide, for 6 months, resulted in partial regression of the two lesions.

**Figure 2:**
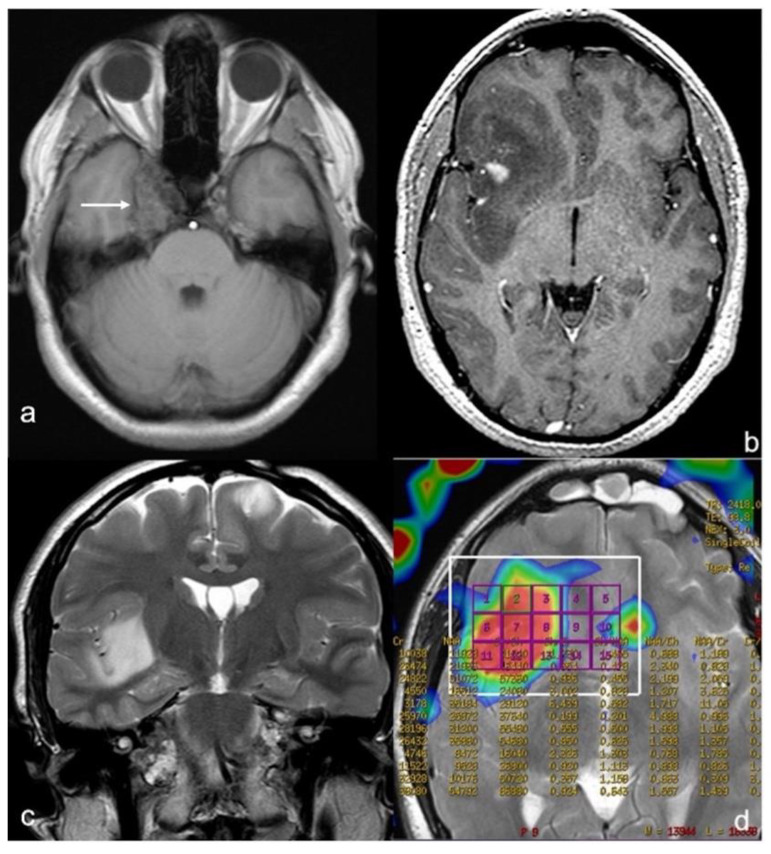
**Brain MRI.** (**a**) **Axial T1 weighted sequence:** bone lesion at right sphenoid wing with irregular borders to refer an enchondroma (with arrow). (**b**) **Post-contrast axial T1-weighted sequence:** multifocal bi-hemispheric lesion, with that one of maximum diameter and with contrast-enhancement at frontal-temporal-insular region of the right hemisphere. (**c**) **Coronal T2 weighted sequence:** multifocal lesion with right insular and left frontal involvement. (**d**) **Perfusion axial sequence:** increased cerebral blood flow at the lesion.

Following treatment, the patient was symptom-free until July 2021, when he experienced left partial motor seizures. A contrast-enhanced brain MRI (Figure 2b,c) showed a multifocal intraparenchymal bi-hemispheric lesion, with the mass of maximum diameter in the frontal-temporal-insular region of the right hemisphere, as well as hypointense in T1- and hyperintense in T2-weighted sequences with deep small areas of contrast enhancement; spectroscopy and perfusion sequences showed increased choline peak, decreased N-acetyl-aspartate peak, and increased blood flow, respectively, at the site of lesion (Figure 2d). The small left frontal lesion was stable. A bone lesion at the right medial sphenoid wing and parasellar region, with the MRI features of an enchondroma, was also evident (Figure 2a).

The detection of a contrast-enhanced area in the latest brain MRI was in favor of tumor progression.

At admission, the neurology examination was normal. The patient underwent a wide microsurgical resection of the frontal-parietal opercular tumor component of the right hemisphere, which appeared as a white-reddish and moderately vascularized mass. The part of the tumor encompassing the M3 branches of middle cerebral artery was left, to avoid postoperative neurological deficits and to preserve the patient quality of life. The component of the tumor that affected the frontal lobe of the left hemisphere was also not removed, because it did not change over time. The postoperative course was uneventful. A contrast-enhanced brain MRI at 48 h showed satisfactory tumor removal of the supra-sylvian component without neurosurgical complications.

### 3.2. Diagnosis

**Figure 3:**
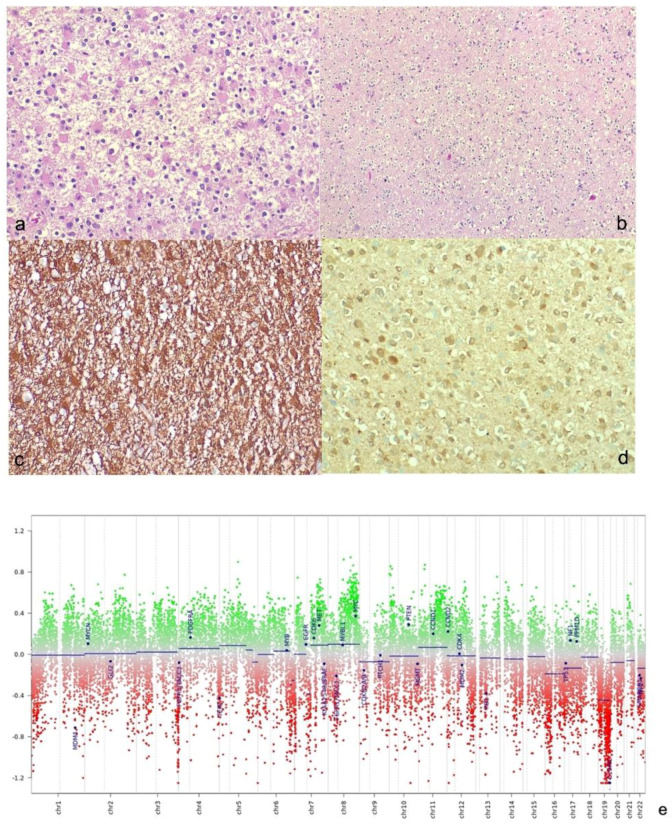
**Histologic and immunohistochemical studies.** (**a**) Neoplasm with fibrillary background, composed of mildly pleomorphic cells having hypercromic and eccentric nuclei and dense cytoplasm with “glassy” appearance (gemistocytes) (*Hematoxylin-eosin staining, original magnifyication 20×*). (**b**) Some fields were oligo-like cells rich (*Hematoxylin-eosin staining, original magnification 10×*). (**c**) Neoplastic cells were diffusely GFAP positive (*Immunoperoxidase staining, original magnification 10×*). (**d**) The immunohistochemical assessment of IDH1 status revealed an IDH-mutant diffuse glioma (*Immunoperoxidase staining, original magnification 20×*). (**e**) Copy Number Variations analysis: The analysis of CNVs showed deletion involved chromosome 19 and partial amplification of MYC. We considered significant a gain of genetic materials if the score, obtained from ratio of case and control sample, was >0.4; as well, we considered significant a loss of genetic materials if the ratio score was <0.4.

The histology showed a tumor with fibrillary background and increased cellularity, with pleomorphic cells but without microvascular proliferation or necrosis; the immunohistochemical study was positive for GFAP and IDH1 R132H mutation. IDH1 mutation was confirmed by DNA sequencing. The mitotic activity was inconsistent, although the proliferation index Ki67-MIB 1 was 15% (Figure 3). The positive methylation status of the MGMT gene promoter was assessed by Methylation Specific PCR (MSP) and confirmed by extrapolation of MGMT methylation status from raw data of EPIC array (Illumina 850k). To assess the codeletion status of 1p19q, we performed Multiplex Ligation Probe Amplification (MLPA): the absence of co-deletion was also confirmed by the analysis of Copy Number Variation (CNVs) extrapolated from METHYLOME raw data (Figure 3e). 

In addition, we analyzed the epigenome profile of the tumor using a previously used bioinformatic tool (DKFZ, Heidelberg, Germany, https://www.dkfz.de/de/index.html, accessed on 7 May 2022). Generated methylation data were compared with the Heidelberg brain tumor classifier to assign a subgroup score for the tumor, compared with 91 different brain tumor entities. The bioinformatic analysis showed a strong match for IDH mutant astrocytoma methylation class [31,32].

The detection of inconsistent mitotic activity allowed us to exclude a high-grade tumor; nevertheless, because of the high value of Ki67, unusual for low-grade gliomas, and the contrast enhancement of the lesion, a typical feature of high-grade gliomas, to further discriminate between high- and low-grade tumors we performed an analysis of two prognostic negative biomarkers implemented in the new classification of tumors of the central nervous system [33]: TERT promoter mutations and CDKN2A/B gene deletion. To detect TERT promoter mutation we assessed direct DNA sequencing, but the analyzed sample did not present promoter mutation. To verify CDKN2A/B gene deletion we analyzed CNVs but, similarly, the deletion was not present, as shown in Figure 3e.

The global histopathological, genomic and epigenomic analysis oriented the diagnosis in favor of WHO grade 2, IDH mutant astrocytoma [34]. 

**Figure 4:**
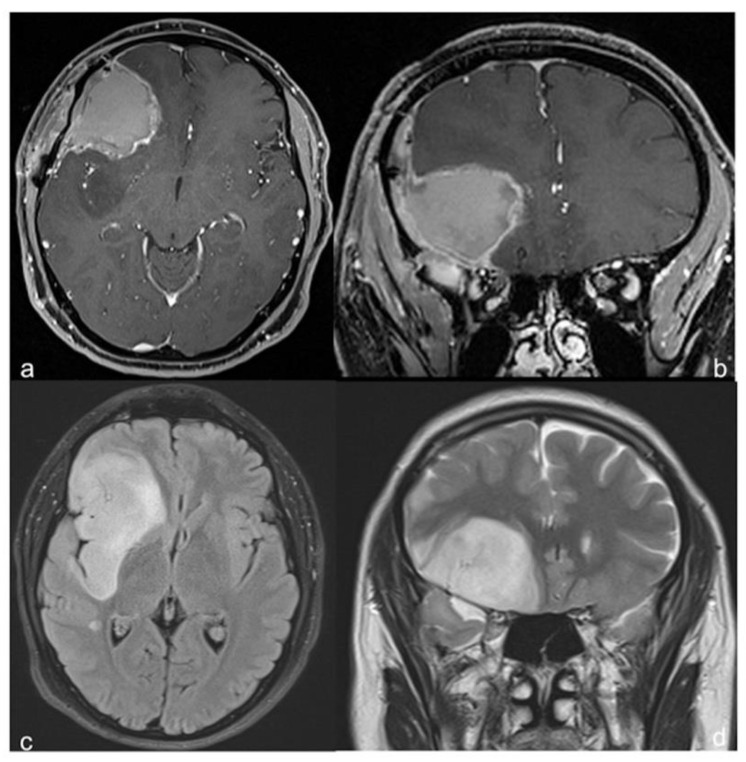
**Postoperative brain MRI**. Axial (**a**) and coronal (**b**) postcontrast T1-weighted, and axial (**c**) and coronal (**d**) T2-weighted, sequences; large resection of the frontal supra-sylvian tumor component.

The contrast-enhanced brain MRI taken one month after surgery confirmed the wide resection of the right frontal-opercular component of the tumor (Figure 4).

The high value of Ki67-Li and the contrast enhancement on MRI witnessed aggressive clinical behavior, so the patient was discharged with indication to perform radiotherapy [35] and a close follow-up through contrast-enhanced brain MRI at 3 months. 

### 3.3. Literature Review

The incidence of brain glioma ranges from 2.4% [18,28] to 16% [18,28] in patients with OD. To the best of our knowledge, only 31 cases, including our own, are reported in the literature (Table 1, Table 2 and Table 3).

Patients were 18 males (60%) and 12 females (40%) with age at diagnosis of the brain glioma ranging from 6 to 55 years (median 26 years) (Table 2). The brain glioma was a single lesion in 16 patients (51%) (mainly with frontal location) and multicentric in 15 (49%) (of which, gliomas were only supratentorial in 2/3 and both supra- and infratentorial in 1/3). The histological study mainly showed astrocytic tumors (62%) and gliomas of WHO grades II and III, whereas only 2 cases out of 29 (7%) were glioblastomas. Among the 10 patients on which biomolecular studies were performed, eight showed positive IDH1 R132H mutations in the brain glioma; in three of them, this mutation was simultaneously detected in the enchondroma.

The management of brain gliomas in the reviewed cases was as follows (Table 3): at the initial diagnosis, 16 patients (12 with multicentric and four with single lesion) underwent a biopsy, six a craniotomy with tumor resection, and three were not operated on; a craniotomy was performed in response to tumor progression in four patients who had undergone a previous biopsy. Radiotherapy (RT), reported in 16 cases, was administered at the initial diagnosis of 12 and at progression in two. Finally, only five patients were treated by chemotherapy.

The outcome was as follows (Table 3): among the 13 patients whose data were available, nine were stable and progression-free at the median follow-up of 15 months, and four experienced progression after a median follow-up of 41 months. Fifteen of eighteen patients with available data were still alive at the median follow-up of 33 months, while three died from 11 to 96 months after the initial diagnosis.

## 4. Discussion

Patients with OD are not only at risk of malignant transformation of enchondromas to chondrosarcomas [1,10,29], with a variable rate from 20 to 50% [1,10,29], but are also at risk of occurrence of other neoplasms, such as gliomas, ovarian tumors [4], pituitary and thyroid adenomas, and leukemia [3]. The prevalence of malignancies in individuals with OD is about 53% [28], among which the most common is represented by chondrosarcoma (30.6%) [28]. The diagnosis of cancer is made at a median age of 26 years (from 1 to 69 years), while the median time lapse between diagnoses of enchondromatosis and malignancy is eleven years [28]. 

The occurrence rate of glioma during the life of these patients ranges from 2.4% [18,28] to 16% [18,28], with only thirty cases reported in literature. 

Prior to 2009, the only approved etiologic risk factors of occurrence of glioma were high-dose radiation and some rare familial cancer syndromes [40]. Since then, 10 inherited variants, around eight genes’ defined risk loci, have been discovered to be associated to an increased risk of glioma’s occurrence [40].

Most sporadic adult diffuse grade II and III gliomas harbor mutations of the IDH1 and/or IDH2 genes, which is considered the first event driving oncogenesis of these tumors [36,41]. 

Ollier disease is due to early post-zygotic IDH mutations, leading to somatic mosaic mutations of IDH1 or IDH2 [38,42]. 

IDH 1 mutations account for amino acid substitution in the active site of the enzyme in codon 132 (R132H), resulting in the abnormal production of 2-hydroxyglutarate, which causes histone and DNA methylation, as well as altered cellular differentiation, hence promoting tumorigenesis. In addition to gliomas, cartilaginous tumors experience IDH mutation [36,37,38,42], but unlike enchondromas, isolated IDH mutation is not enough to induce glioma genesis; further mutations, such as ATRX and/or TP53, are needed [41].

Compared to the sporadic forms of IDH mutant gliomas, gliomas occurring in OD patients are also mainly diffuse and low-grade, and more frequently involve frontal lobes; conversely, they are diagnosed at an earlier age (26 vs. 44 years) and show higher rates of multicentric lesions (49% in our review) and brainstem localization (29%), and significantly lower rate of glioblastomas histotype (7%).

In cartilaginous tumors of patients with enchondromatosis, the expression of the IDH1 R132C mutation is more common than IDH1 R132H (70% vs. 15%, respectively) [38,42], whereas IDH1 R132H represents the main mutation (90%) in sporadic IDH mutant gliomas [36].

Only 30 cases of gliomas associated to Ollier disease have been reported in the literature, and most of these belong to the pre-molecular era; therefore, only in seven studies [10,14,23,24,30], our own included, for a total of ten patients, the molecular analysis of gliomas has been performed (Table 1): eight of these (80%) presented the IDH1 R132H mutation. Furthermore, only in three cases [23,30], our own included, molecular analysis was performed in both an enchondroma and glioma, and in all three cases (100%) the identical IDH1 R132H mutation was detected in both tumors. 

Although from a small sample, these data suggest that patients with Ollier disease harboring the IDH1 R132H mutation in enchondroma are at major risk of developing gliomas, compared to enchondromatosis patients with the IDH R132C mutation in enchondroma. Furthermore, the early acquisition of IDH mutation accounts for the occurrence of glioma at a younger age in these patients. Therefore, once a diagnosis of OD with IDH R132H mutation is made, we consider it be useful to perform close surveillance through brain contrast-enhanced MRI, to detect possible early stage gliomas, when still asymptomatic, of small size, without compromission of the neurological functional areas nor vital neurovascular structures, so to plan the best tailored management. The incidence of postoperative complications and seizures is higher in patients with symptomatic compared to those with incidental low-grade glioma [43] and the overall survival (OS) of incidental low-grade glioma (LGG) patients treated with surgery is better than that of symptomatic LGG patients [39,44].

The median silent phase of glioma, defined as the time from the generation of the tumor to the occurrence of tumor-related symptoms, was estimated by Pallud et al. to be about 12 years (range 1.6–39.4 years) [45], and the average growth rate of LGG is 2.7 mm/year (range from 1 to 5 mm) [43].

The rate of malignant transformation of LGG ranges from 25% to 72% [46] and it is influenced by several risk factors, such as preoperative tumor size, velocity of diametric expansion, astrocytoma histology, extent of resection, and treatment with chemotherapy, radiotherapy, and adjuvant chemo-radiotherapy [46]. 

Being the extent of resection the main predictive risk factor of progression free-survival (PFS), overall survival and malignant progression free-survival (MPFS) in glioma patients [43], the possibility to detect a glioma in early stage, when of a small size, should allow to achieve the maximal safe tumor resection preserving the patient’s quality of life.

## 5. Conclusions

We conclude that patients with Ollier disease which harbor the IDH1 R132H mutation in enchondroma could be at major risk to develop gliomas, compared to those with the IDH1 R132C mutation. Therefore, we suggest routine performance of molecular analysis of cartilaginous tumors in patients with OD, and, if IDH1 132H mutation is detected, performance of close surveillance through periodical ambulatorial and radiological control with contrast-enhanced MRI of the brain, to detect the eventual CNS tumor when asymptomatic and of small size, to plan the best management.

## Figures and Tables

**Table 1:** Demographic and pathological data.

N. of Cases	Authors/Year	Sex/Age	CNS Tumor		Molecular Analysis		Surgery at Initial Diagnosis	Adjuvant Treatment	Progression/Recurrence	Outcome
			Histology	Location	Glioma	Enchon-Droma				
1	Becker et al. [7]. 1979	n.s./26	Oligoastrocytoma (grade II)	r. frontal	n.a.	n.a.	n.s.	n.s.	n.s.	n.s.
2	Rawlings et al. [34]. 1987	M/29	anaplastic astrocytoma (grade III)	multicentric (r. cerebellum, r. frontal)	n.a.	n.a.	biopsy	RT	n.s.	n.s.
3	Mellon et al. [25]. 1988	M/34	astrocytoma (grade II)	r. frontal	n.a.	n.a.	craniotomy	n.s.	n.s.	n.s.
4	Schwartz et al. [36]. 1987	M/38	malignant astrocytoma (grade III)	temporo- parietal	n.a.	n.a.	n.s.	n.s.	n.s.	dead
5	Patt et al. [30]. 1990	M/24	Astrocytoma (grade II)	brainstem	n.a.	n.a.	biopsy	n.s.	n.s.	n.s.
6	Bendel et al. [8]. 1991	F/29	high grade astrocytoma (grade III)	l. frontal	n.a.	n.a.	n.s.	n.s.	n.s.	n.s.
7	Chang et al. [12]. 1994	M/23	anaplastic astrocytoma (grade III)	Multicentric (both hemispheres)	n.a.	n.a.	biopsy	WBRT	no at 3 y	Alive at 3 y
8	Chang et al. [12]. 1994	M/25	oligodendroglioma	r. frontal	n.a.	n.a.	Craniotomy (STR)	RT	no at 8 mo.	Alive at 8 mo
9	Chang et al. [12]. 1994	M/46	oligoastrocytoma	Multicentric (both frontal lobes)	n.a.	n.a.	biopsy	RT	no at 4 mo.	Alive at 4 mo.
10	Hofman et al. [18]. 1998	M/28	Astrocytoma (grade II)	multicentric (l. temporal, brainstem)	n.a.	n.a.	biopsy	RT	no at 1 y	Alive at 1 y
11	Balcer et al. [5]. 1999	F/23	astrocytoma	pons	n.a.	n.a.	none	RT	n.s.	n.s.
12	Frappaz et al. [16]. 1999	M/16	astrocytoma	brainstem	n.a.	n.a.	none	RT	no at 7 mo.	Alive at 7 mo.
13	Van Nielen et al. [37]. 1999	M/28	Astrocytoma (grade II)	multicentric (l. temporal, brainstem)	n.a.	n.a.	biopsy	RT	n.s.	n.s.
14	Simsek et al. [38]. 2002	F/7	Astrocytoma (grade II)	r. frontal	n.a.	n.a.	n.s.	n.s.	n.s.	n.s.
15	Mahafza et al. [24]. 2004	F/21	Astrocytoma (grade II)	r. frontal, brainstem	n.a.	n.a.	biopsy	n.s.	n.s.	n.s.
16	Koc et al. [21]. 2006	F/28	anaplastic oligoastrocytoma	r. frontal	n.a.	n.a.	craniotomy	RT at recurrence	recurrence at 6 y, reoperation	alive at 10 y
			(grade III)							
17	Ranger et al. [33]. 2009	F/6	glioblastoma	l. thalamus	n.a.	n.a.	biopsy	RT + CHT	progression	dead at 11 mo
18	Walid et al. [39]. 2008	M/14	anaplastic astrocytoma (grade III)	cerebellum	n.a.	n.a.	n.s.	n.s.	n.s.	n.s.
19	Hori et al. [19]. 2010	M/19	anaplastic astrocytoma (grade III)	Multicentric (brain, brainstem)	n.a.	n.a.	craniotomy	RT + CHT (TMZ)	stable	alive
20	Bathla et al. [6]. 2012	M/16	Astrocytoma (grade II)	multicentric (both frontal parietal lobes)	IDH1 R132H	n.a.	biopsy	CT and craniotomy at progression	progression to anaplastic astrocytoma (grade III) at 3 y, reoperation	Dead at 8 y
21	Pearce et al. [31]. 2012	M/19	Oligoastrocytoma (grade II)	multicentric (both hemispheres)	no mutation	n.a.	biopsy	no	progression at 9 mo. after biopsy, craniotomy	n.s.
22	Gajavelli et al. [17]. 2016	F/55	anaplastic astrocytoma (grade III)	multicentric (l. frontal, temporal, parietal)	IDH1 R132H	n.a.	craniotomy(STR)	no	stable at 3 y	alive at 3 y
23	Bonnet et al. [9]. 2016	F/28	Oligoastrocytoma (grade II)	multicentric	IDH1 R132H	n.a	biopsy	n.s.	n.s.	alive at 2.5 y
				(temporal, frontal)						
24	Bonnet et al. [9]. 2016	M/26	n.s.	frontal	n.s.	n.a	biopsy	n.s.	n.s.	alive at 1 y
25	Bonnet et al. [9]. 2016	F/30	Oligodendroglioma (grade II)	multicentric (frontal, temporal	IDH1 R132H	n.a	biopsy	n.s.	n.s.	Alive at 4 y
26	Bonnet et al. [9]. 2016	M/31	Glioblastoma (grade IV)	multicentric (frontal, parietal	IDH1 R132H	n.a	biopsy	n.s.	n.s.	alive at 9 mo
27	Bonnet et al. [9]. 2016	F/31	Oligoastrocytoma (grade III)	frontal	IDH1 R132H	IDH1 R132H	biopsy	n.s.	n.s.	alive at 1.5 y
28	Achiha et al. [1]. 2017	M/32	oligodendroglioma	l. frontal	IDH1 R132H	IDH1 R132H	n.s.	n.s.	n.s.	n.s.
29	Al Rumeh et al. [2]. 2020	F/23	diffuse astrocytoma (grade II)	multicentric (l. frontal)	IDH1 R132C	n.a.	craniotomy	n.s.	n.s.	n.s.
30	Karabulut et al. [20]. 2021	F/23	diffuse midline glioma	pons	n.a.	n.a.	none	RT + CHT	stable	alive
31	Present case	M/33	diffuse astrocytoma (grade II)	multicentric (both hemispheres)	IDH1 R132H	IDH1 R132H	biopsy	RT + CHT (TMZ) at initial diagnosis	progression at 48 mo. after biopsy, reoperation + RT	alive at 5 y

n.s.: not specified; l.: left, r.: right; n.a.: not available; RT: Radiotherapy; CHT: Chemotherapy; TMZ: Temozolomide; y: years; mo.: months; STR: Sub Total Resection.

**Table 2:** Summary of the epidemiological and pathological data of the 31 reported cases of OD with brain glioma.

Covariates	Overall Series
	(31 Patients)
**Age**	**6–55 y**
	(median 26 y)
**Sex**	30 *
F	12 (40%)
M	18 (60%)
**Location**	31 *
***Single lesion***	16 (51%)
supratentorial	11
brainstem	4
cerebellar	1
***Multicentric***	15 (49%)
only supratentorial	10
supra- and infratentorial	5
**Pathology**	
***WHO grade***	24 *
II	13
III	9
IV	2
***Histological type***	29 *
astrocytic	18
oligodendroglial	3
oligo-astrocytic	6
glioblastoma	2
**Molecular biology**	10 * gliomas
***Detection of IDH1 R132H mutation:***	3 * enchondromas
glioma	8/10
enchondroma	3/3

* Cases with specified data; M: Male; F: Female.

**Table 3:** Summary of the data on management and outcome of the 31 reported cases of OD with brain glioma.

Covariates	Overall Series
	(31 Patients)
**Surgery**	25 *
biopsy	16
craniotomy	6
none	3
**Radiotherapy**	16 *
at initial diagnosis	12
at progression	2
none	2
**Chemotherapy**	7 *
at initial diagnosis	3
at progression	2
none	2
**Glioma evolution**	13 *
Stable	9 (from 3 to 36 mo.)
Progression	4 (from 9 to 72 mo.)
**Outcome**	18 *
Alive	15 (from 4 to 187 mo.)
Dead	3 (from 11 to 96 mo.)

* Cases with specified data.

## Limitations of the Study

Although the literature review is wide and accurate, the sample of cases is rather small, due to the rarity of this pathological entity. Besides, many reviewed studies do not include complete data on several aspects, mainly concerning the molecular aspects.

## Advantages

The purpose of the study to establish a diagnostic protocol to make an early and prompt diagnosis of glioma associated to Ollier Disease, would allow to plan the best management for these patients improving their overall survival and quality of life. Further studies are needed to confirm our hypothesis.

## Abbreviations

OD	Ollier Disease
CT	Computed Tomography
MRI	Magnetic Resonance Imaging
WHO	World Health Organization
CNS	Central Nervous System
PFS	Progression Free Survival
OS	Overall Survival
MPFS	Malignant Progression-Free Survival
